# Morphometric analysis of extraocular muscles and proptosis by computed tomography in Graves’ orbitopathy

**DOI:** 10.1590/0100-3984.2024.0040-en

**Published:** 2024-12-27

**Authors:** Erika Mayumi Watanabe, Ronan Yudi Cavazzana, Douglas de Aguiar Manso Ribeiro, Lorena Candido Brandão, Ana Victória Haddad, José Eduardo Corrente, André Petean Trindade, Eliane Chaves Jorge

**Affiliations:** 1 Faculdade de Medicina de Botucatu, Universidade Estadual Paulista “Júlio de Mesquita Filho” (Unesp), Botucatu, SP, Brazil

**Keywords:** Graves ophthalmopathy, Tomography, X-ray computed, Oculomotor muscles, Exophthalmos, Oftalmopatia de Graves, Tomografia computadorizada por raios X, Músculos oculomotores, Exoftalmia

## Abstract

**Objective:**

To assess the prevalence of changes on computed tomography (CT) in Graves’ orbitopathy (GO) and to correlate those changes with disease activity, as well as with clinical and biochemical variables.

**Materials and Methods:**

This was a retrospective study, conducted at a tertiary hospital, of clinical, biochemical, and imaging data from consecutive patients with GO who underwent at least one orbital CT scan between July 2012 and December 2020. A single observer quantified the thickness of the extraocular muscles and the degree of proptosis. Clinical and biochemical variables were analyzed to determine whether they correlated with CT changes, GO activity, and GO severity.

**Results:**

Our sample included data from 67 patients with GO (134 orbits), 50 (74.6%) of whom were female. There were positive correlations between the clinical activity score and increase in thyroid-stimulating factor/free thyroxine, between the severity of GO and the increase in the thickness of the extraocular muscles, and between the degree of proptosis and muscle thickness.

**Conclusion:**

Orbital CT proved effective in detecting thickening of the extraocular muscles and proptosis in patients with GO, changes that correlated significantly with clinical and biochemical variables. Muscle thickening was associated with the severity of GO and could be a biomarker of the risk of vision loss.

## Abstract (pt)

**Objetivo:**

Conhecer a prevalência de alterações tomográficas na orbitopatia de Graves (OG) e correlacioná-las com atividade da doença e variáveis clínico-laboratoriais.

**Materiais e Métodos:**

Estudo retrospectivo, em hospital terciário, com dados clínico-laboratoriais e tomográficos de pacientes consecutivos portadores de OG, com pelo menos uma tomografia computadorizada de órbitas entre julho de 2012 e dezembro de 2020. Um único examinador quantificou a espessura da musculatura ocular extrínseca e o grau de proptose. Variáveis clínicolaboratoriais foram correlacionadas com alterações tomográficas, atividade e gravidade da OG.

**Resultados:**

Foram incluídos 67 pacientes com OG (134 órbitas), sendo 50 do gênero feminino (74,6%). Houve correlação positiva entre o escore de atividade clínica e aumento de TSH/T4 livre, entre a gravidade da OG e aumento da espessura da musculatura extrínseca, e entre o grau de proptose e espessamento muscular.

**Conclusão:**

A tomografia computadorizada de órbitas se mostrou efetiva na detecção do espessamento da musculatura ocular extrínseca e da proptose em pacientes com OG, alterações que se correlacionaram de forma significativa com variáveis clínicas e bioquímicas. O espessamento muscular se associou com a gravidade da OG e pode ser um biomarcador de risco de perda da visão.

## INTRODUCTION

Graves’ orbitopathy (GO), or dysthyroid orbitopathy, is the most relevant extrathyroidal manifestation of Graves’ disease. It is an autoimmune inflammatory condition that leads to remodeling of the orbital tissues and hypertrophy of the extraocular muscles; it can cause mild changes in the ocular surface, eyelid retraction, proptosis, and even permanent loss of vision due to optic neuropathy^(1,2)^.

The diagnosis of GO is based on clinical findings and laboratory tests. However, imaging examinations are vital to exclude differential diagnoses, such as orbital tumors, evaluate the initial orbital changes, assess disease progression, and plan treatments and surgical interventions^(3,4)^.

The choice of the appropriate imaging test should consider availability, cost, and burden to the patient. Although practical and economical, ocular ultrasound does not allow a detailed assessment of the extraocular muscles. Although magnetic resonance imaging is more accurate for the volumetric evaluation of tissues and inflammatory activity, it is costly and, in Brazil, is still difficult to access via the Brazilian Unified Health Care System^(5,6)^. Computed tomography (CT) of the orbits is a practical, cost-effective examination that allows assessment of the bony anatomy of the orbit and measurements of the extraocular muscles, which aids in the early detection of GO, even before the clinical signs of the disease appear^(7,8)^. However, there have been few studies aimed at validating CT for use in diagnosing and managing GO.

The aim of the present study was to employ CT in determining the prevalence of ocular alterations in patients with GO, correlating the CT findings with clinical data, biochemical data, and GO activity.

## MATERIALS AND METHODS

This was a retrospective, single-center study conducted at a tertiary care hospital that included consecutive patients ≥ 18 years of age with GO, diagnosed according to the criteria established by Bartley et al.^(9)^, in different stages of the disease. The patients were monitored regularly by a team of ophthalmologists and endocrinologists, with complete clinical and biochemical data and at least one orbital CT examination performed between July 2012 and December 2020. The study was approved by the Research Ethics Committee of the Faculdade de Medicina de Botucatu – Universidade Estadual Paulista “Júlio de Mesquita Filho” (Reference no. 3556843). Because of the retrospective nature of the study, the requirement for informed consent was waived.

Patients for whom there were no available clinical data or laboratory test results during the study period were excluded, as were those who had not undergone orbital CT during the study period and those who had undergone CT at another facility.

At the time of the CT scan, we collected data on disease activity—according to the criteria of the clinical activity score (CAS) and the European Group on Graves’ Orbitopathy (EUGOGO)—the thyroid-stimulating factor (TSH) level, and the free thyroxine (T4) level, categorizing the state as hyperthyroidism, hypothyroidism, or euthyroidism.

All images were acquired in one of two multidetector CT scanners—Activision 16 (Toshiba Medical Systems Corporation, Otawara, Japan) or Optima 64 (GE Health-Care, Arlington Heights, IL, USA)–with multiplanar reconstruction, using the orbit protocol, with a dedicated field of view, a slice thickness of 1.0–0.5 mm and an interslice gap of 0.6–0.5 mm, with modulated tube voltage and current.

From the CT images, a morphometric analysis of the extraocular muscles and an evaluation of the degree of proptosis of the orbits were performed by the same examiner, who was blinded to the clinical and biochemical data.

**Figure 1.**
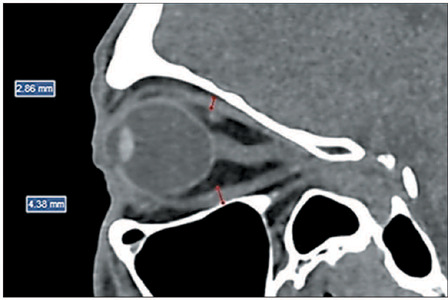
CT of orbits in the sagittal plane: measurement of the thickness of the muscle bellies of the SC and IR.

**Figure 2.**
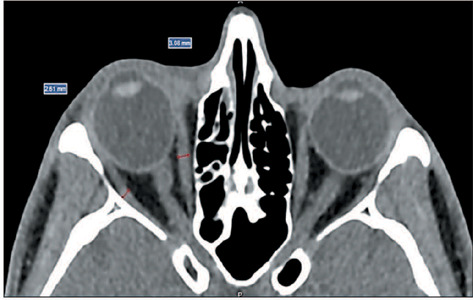
CT of orbits in the axial plane: measurement of the thickness of the muscle bellies of the MR and LR.

For measurements of the thickness of the extraocular muscles, the standards employed were those established by Ozgen and Ariyurek^(10)^ and subsequently adapted by Machado et al.^(11)^ The inferior rectus muscle (IR) and the superior complex (SC), composed of the superior rectus muscle (SR) and the levator palpebrae superioris muscle, were measured in the sagittal plane (Figure 1), whereas the medial rectus muscle (MR) and the lateral rectus muscle (LR) were measured in the axial plane (Figure 2).

**Figure 3.**
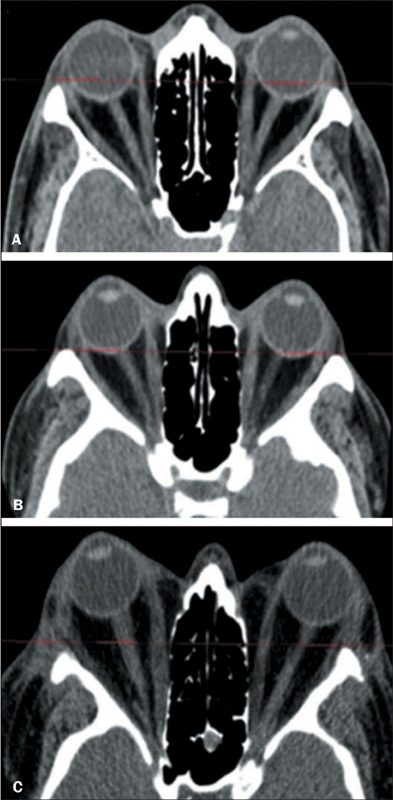
CT scan of orbits in the axial plane: degree of proptosis. **A:** Grade 1 proptosis. **B:** Grade 2 proptosis. **C:** Grade 3 proptosis.

The position of the globe was calculated by determining the distance between the interzygomatic line and the posterior margin of the ocular globe at its central aspect, in the axial plane. Proptosis was classified in degrees (Figure 3) according to the criteria established by Morax et al.^(12)^.

The outcomes evaluated were the activity/severity of GO, quantified by calculating the CAS and determining the EUGOGO classification; the correlation between TSH and CT findings; the correlation between free T4 and CT findings; a change in the thickness of the extraocular muscles; and a change in the position of the eyeball (proptosis).

The statistical analysis was performed with the Statistical Analysis System for Windows, version 9.4 (SAS Institute Inc., Cary, NC, USA). Thickness variables were categorized according to the reference standard. The associations that tomographic and clinical variables showed with disease severity and activity were determined with the chi-square test. Means and standard deviations were calculated to assess the normality of the thickness variables. In all analyses, the significance level was set at 5% or the corresponding p-value.

## RESULTS

### Clinical and laboratory evaluation

Data from 67 patients (134 orbits) diagnosed with GO at different stages of the disease were included, of whom 50 (74.6%) were female. Patient ages ranged from 21 to 84 years, with a mean of 48 ± 14.7 years. Fifteen patients (22.4%) had diabetes, and 13 (19.4%) were current smokers.

According to the CAS, 61 patients (91%) were in the inactive phase of GO at the time of the CT scan. According to the EUGOGO severity criteria, 52 patients (77.6%) had mild GO, 14 (20.9%) had moderate-to-severe GO, and one (1.5%) had very severe GO.

The EUGOGO severity class did not correlate significantly with sex (*p* = 0.4213) or age (*p* = 0.5527).

According to the levels of TSH and free T4, the status was categorized as euthyroidism in 40 patients (60.6%), hyperthyroidism in 18 (27.3%), and hypothyroidism in eight (12.1%). Although there was a positive correlation between altered TSH/free T4 levels and the CAS (*p* = 0.0052), those levels did not correlate significantly with the EUGOGO severity classification (*p* = 0.742).

### Morphometric evaluation of extraocular muscles

The data regarding the general thickness measurements of the extraocular muscles MR, LR, and IR, as well as those of the SC, are presented in Table 1. In both eyes, the mean values were highest for the IR: 5.79 mm in the right eye (oculus dexter–OD) and 5.51 mm in the left eye (oculus sinister–OS). However, the proportion of cases in which a change in thickness was more significant for the LR: 79.1% in the OD and 76.1% in the OS.

**Table 1:** Thickness values (mm) of the extraocular muscles in 67 patients (134 orbits) with GO.

Muscle	Reference Mean* (mm)	Mean (mm)	SD (mm)	Min. (mm)	Max. (mm)	Median (mm)	Frequency	
							Normal n (%)	Altered n (%)
MR OD	4.2	5.08	1.26	3.2	9.7	4.9	40 (59.7)	27 (40.3)
LR OD	3.3	5.12	1.28	2.2	9.7	5.1	14 (20.9)	53 (79.1)
IR OD	4.8	5.79	1.76	2.8	13.0	5.4	44 (65.7)	23 (34.3)
SC OD	4.6	4.69	1.41	2.3	8.7	4.6	52 (77.6)	15 (22.4)
MR OS	4.2	4.86	1.22	2.1	9.6	4.8	40 (59.7)	27 (40.3)
LR OS	3.3	4.94	1.17	2.4	8.4	4.7	16 (23.9)	51 (76.1)
IR OS	4.8	5.51	1.88	2.3	12.8	5.0	46 (68.6)	21 (31.4)
SC OS	4.6	4.59	1.34	2.3	7.7	5.4	51 (76.1)	16 (23.9)

* Reference means: Ozgen and Ariyurek^(10)^, adapted by Machado et al.^(10)^.

SD, standard deviation; Min., minimum; Max., maximum.

Table 2 shows the mean values of extraocular muscle thickness on the 67 CT examinations analyzed. The IR presented the highest means on the examinations that showed alterations from GO, followed by the SC, MR, and LR in both eyes. The sequence was IR, MR, SC, and LR in normal volumetric analysis. The mean values of muscle thickness in the studied population were as follows: MR (OD = 4.3 mm and OS = 4.1 mm), LR (OD = 3.5 mm and OS = 3.6 mm), IR (OD = 4.8 mm and OS = 4.6 mm), SC (OD = 4.1 mm and OS = 4.0 mm). The MR and IR values were close to the international reference values, whereas the LR values were higher and the SC values were lower^(10)^.

**Table 2:** Comparison between the mean values for thickness of the extraocular muscles, considering the 67 CT examinations (134 orbits), by whether the measurements were categorized as normal or altered.

Muscle	Measurements	CTs (n)	Mean (mm)	SD (mm)	Min. (mm)	Max. (mm)	Median (mm)	95% CI (mm)
MR OD	Altered	27	6.19	1.23	4.6	9.7	5.8	5.70–6.67
	Normal	40	4.34	0.52	3.2	5.1	4.4	4.17–4.51
LR OD	Altered	53	5.54	1.06	4.0	9.7	5.3	5.25–5.83
	Normal	14	3.52	0.62	2.2	4.4	3.6	3.16–3.88
IR OD	Altered	23	7.58	1.80	5.5	13.0	7.2	6.80–8.36
	Normal	44	4.85	0.70	2.8	5.8	4.95	4.64–5.06
SC OD	Altered	15	6.75	0.97	5.6	8.7	6.3	6.22–7.29
	Normal	52	4.10	0.85	2.3	5.6	4.3	3.86–-4.33
MR OS	Altered	27	5.96	1.04	5.0	9.6	5.6	5.54–6.37
	Normal	40	4.13	0.66	2.1	5.3	4.25	3.92–4.34
LR OS	Altered	51	5.35	0.99	4.2	8.4	5.0	5.07–5.63
	Normal	16	3.63	0.57	2.4	4.4	3.7	3.33–3.94
IR OS	Altered	21	7.49	2.08	5.5	12.8	6.9	6.54–8.43
	Normal	46	4.61	0.77	2.3	6.0	4.7	4.38–4.84
SC OS	Altered	16	6.48	0.63	5.6	7.7	6.4	6.14–6.81
	Normal	51	4.0	0.88	2.3	6.0	4.1	3.76–4.25

SD, standard deviation; Min., minimum; Max., maximum; CI, confidence interval.

Among the 90 eyes (180 orbits) evaluated by CT in the 67 patients in our sample as a whole, the altered thickness values were similar to those obtained for the subsample in which we considered only one examination per patient and followed the sequence IR, SC, MR, and LR. In the normal examinations, the slight difference in the size of the sample (13 eyes; 26 orbits) did not alter the values found for the MR (OD = 4.3 mm and OS = 4.1 mm), IR (OD = 4.8 mm and OS = 4.6 mm), or SC (OD = 4.1 mm and OS = 4 mm). The LR values were minimally higher (OD = 3.6 mm and OS = 3.7 mm).

The correlation between thickness values and GO activity did not show statistical significance. However, the thickness values did show a positive correlation with the EUGOGO disease severity classification (Table 3).

**Table 3:** Correlation between the thickness values of the extraocular muscles and the EUGOGO severity classification in 90 CT examinations (180 orbits).

Muscle	Thickness (mm)	EUGOGO classification		Total (n)	P
		Mild n (%)	Moderate/Severe n (%)		
MR OD	Altered	20 (32.3)	17 (60.7)	37	0.0111
	Normal	42 (67.7)	11 (39.3)	53	
LR OD	Altered	45 (72.6)	22 (78.6)	67	0.5464
	Normal	17 (27.4)	6 (21.4)	23	
IR OD	Altered	18 (29.0)	16 (57.1)	34	0.0109
	Normal	44 (71.0)	12 (42.9)	56	
SC OD	Altered	9 (14.5)	12 (42.9)	21	0.0033
	Normal	53 (85.5)	16 (57.1)	69	
MR OS	Altered	20 (32.3)	17 (60.7)	37	0.0111
	Normal	42 (67.7)	11 (39.3)	53	
LR OS	Altered	43 (69.4)	19 (67.9)	62	0.887
	Normal	19 (30.6)	9 (32.1)	28	
IR OS	Altered	15 (24.2)	15 (53.6)	30	0.0062
	Normal	47 (75.8)	13 (46.4)	60	
SC OS	Altered	9 (14.5)	15 (53.6)	24	0.0001
	Normal	53 (85.5)	13 (46.4)	66	

### Assessment of the globe position

**Figure 4.**
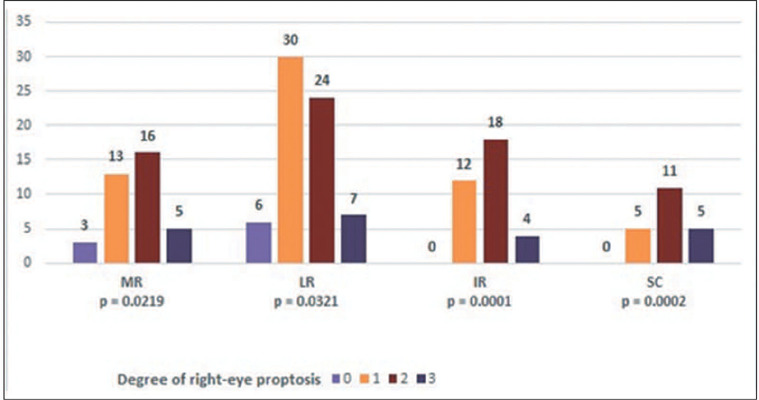
Correlation between the degree of proptosis and thickening of the extraocular muscles in ODs.

**Figure 5.**
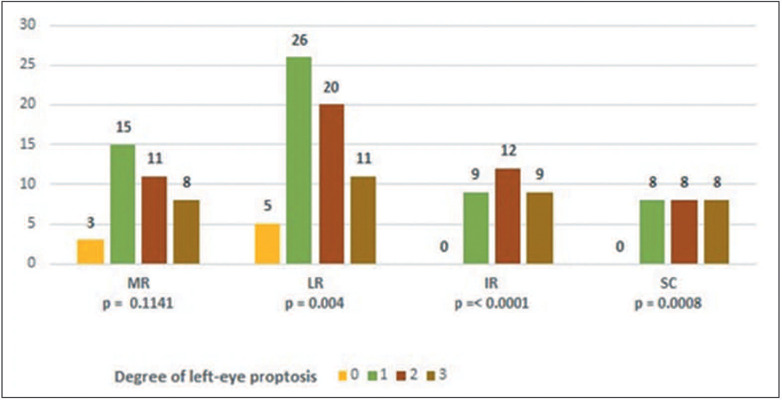
Correlation between the degree of proptosis and thickening of the extraocular muscles in OSs.

The correlation analyses between proptosis and the CAS/EUGOGO criteria revealed no statistically significant differences. However, there was a positive correlation between proptosis and thickening of the extraocular muscles in both eyes, except for the MR in the right eye (Figures 4 and 5). The zero values make it difficult to estimate the effect of the statistical significance of IR and SC.

## DISCUSSION

Imaging examinations allow early detection of GO, even before the appearance of clinical and biochemical changes^(13,14,15)^. Because it is an accessible imaging modality that enables the evaluation of changes in the extraocular muscles, proptosis, and signs of compression of the optic nerve^(7,8)^, CT is an essential tool for diagnosing and monitoring orbitopathy.

In the present study, the prevalence of alterations seen on CT in at least one eye was high (88.05% for proptosis and 80.59% for a change in muscle thickness). Of the patients in our sample, 91% presented no orbitopathy activity at the time of the CT examination, and 60.6% were categorized as being in a state of euthyroidism. These findings confirm that GO presents an evolution independent of the activity of Graves’ disease and that stable thyroid function does not interfere with the regression or stabilization of the ocular condition^(16,17)^.

Among the CT examinations considered normal in our sample, the extraocular muscle thickness values were within the international reference range established in 1988 by Ozgen and Ariyurek^(10)^, based on their analysis of a sample of patients in Turkey. There is no reference table with measurements that represent the normal values for the Brazilian population. The IR presented the highest mean thickness by the reference standard^(10)^. Among the CT examinations that showed alterations, the IR also presented the highest mean values, followed by the SC, MR, and LR. Although this sequence of thickening differs from that found in the literature, which is IR, MR, SR, LR, and the oblique muscles (easily remembered by the mnemonic “I’M SLOw”)^(18)^, it is in agreement with the results of previous studies that compared the increase in thickness with the degree of proptosis^(16,19)^. According to those studies, the IR-MR-SR-LR sequence would occur in grade 2 and grade 3 proptosis, whereas in grade 1 proptosis, the SC would be more affected than the MR.

In our sample, it was impossible to demonstrate a positive correlation between the activity of the orbitopathy and the increase in muscle thickness, as has been shown in other studies^(20,21)^. The probable reason for this finding is that the vast majority of our patients were in a state of euthyroidism and showed no orbital inflammatory activity at the time of the CT examination. However, there was a positive correlation between the disease severity and the increase in muscle thickness, except for that of the LR muscle. The small number of patients with severe or very severe GO in our population could explain that finding, given that the involvement of the LR typically occurs late in the course of GO^(22)^.

In our study, the most common grade of proptosis was grade 1, with a frequency of 43.3% in the right orbit and 46.3% in the left orbit. The proptosis was unilateral in 8% of the patients and asymmetric in 10%. Among patients with GO, studies have estimated the prevalence of unilateral disease to be between 4.5% and 14.0%^(23,24,25,26,27)^, whereas the estimated prevalence of asymmetric disease is 9–34%^(23,24,25,26,27,28,29)^. Current evidence suggests that patients with asymmetric or unilateral GO can progress to bilateral involvement and greater disease severity^(23,24,25,26,27,28,29)^. In daily clinical practice, unilateral involvement in GO must be differentiated from that attributable to other diseases that affect only one eye, and orbital CT can facilitate that differentiation^(30)^.

In the present study sample, proptosis did not correlate with GO activity or severity. Strianese et al.^(31)^ also found no relationship between the CAS and the degree of proptosis in patients with GO. According to those authors, the degree of proptosis is not an appropriate criterion for monitoring asymmetric cases. In their correlation analyses between proptosis and muscle thickening, there was a positive correlation, except for the MR, in the left eyes of the patients studied. Other studies have suggested that the severity of proptosis is more related to the increase in adipose tissue than to muscle thickening^(32,33)^. However, Abreu^(34)^ suggested that expansion of the muscle bellies in the posterior region of the orbit could facilitate venous ectasia at the apex, perpetuating proptosis and congestion, potentially causing glaucoma secondary to trabecular meshwork dysfunction, neuropathy due to optic nerve compression, or permanent vision loss.

Our study has some limitations. The patient sample was relatively small, and all of the patients were evaluated at the same center, a university hospital. There could also have been selection bias due to the retrospective nature of the study. Multicenter studies with larger sample sizes could increase the reliability of the results. Another limitation of our study was that all orbital measurements were performed by the same examiner, which precluded any assessment of interobserver agreement and accuracy.

In conclusion, CT proved to be a sensitive, effective tool for detecting early orbital changes resulting from GO, even in euthyroid patients without orbital inflammatory activity. In patients with GO, extraocular muscle thickening appears to correlate with disease severity and could be a biomarker of the risk of visual loss.

## References

[1] Bartalena L, Tanda ML (2022). Current concepts regarding Graves’ orbitopathy. J Intern Med.

[2] Saeed P, Tavakoli Rad S, Bisschop PHLT (2018). Dysthyroid optic neuropathy. Ophthalmic Plast Reconstr Surg.

[3] Edmunds MR, Boelaert K (2019). Knowledge of thyroid eye disease in Graves’ disease patients with
and without orbitopathy. Thyroid.

[4] Luccas R, Riguetto CM, Alves M (2024). Computed tomography and magnetic resonance imaging approaches to
Graves’ ophthalmopathy: a narrative review. Front Endocrinol (Lausanne).

[5] Shen J, Jiang W, Luo Y (2018). Establishment of magnetic resonance imaging 3D reconstruction
technology of orbital soft tissue and its preliminary application in
patients with thyroid-associated ophthalmopathy. Clin Endocrinol (Oxf).

[6] Rocha AS, Cabral PG, Souza GD (2018). Aspectos radiológicos na avaliação da
oftalmopatia de Graves: uma revisão de literatura. Rev Med Saude Brasilia.

[7] Bradley EA (2001). Graves ophthalmopathy. Curr Opin Ophthalmol.

[8] Weiler DL (2017). Thyroid eye disease: a review. Clin Exp Optom.

[9] Bartley GB, Gorman CA (1995). Diagnostic criteria for Graves’ ophthalmopathy. Am J Ophthalmol.

[10] Ozgen A, Ariyurek M (1998). Normative measurements of orbital structures using
CT. AJR Am J Roentgenol.

[11] Machado KFS, Garcia MM (2009). Oftalmopatia tireoidea revisitada. Radiol Bras.

[12] Morax S, Hamedani M (2000). Exophtalmie. Orientation diagnostique. Rev Prat.

[13] Teng CS, Yeo PP (1977). Ophthalmic Graves” disease: natural history and detailed thyroid
functions studies. Br Med J.

[14] Salvi M, Zhang ZG, Haegert D (1990). Patients with endocrine ophthalmopathy not associated with overt
thyroid disease have multiple thyroid immunological
abnormalities. J Clin Endocrinol Metab.

[15] Enzmann DR, Donaldson SS, Kriss JP (1979). Appearance of Graves’ disease on orbital computed
tomography. J Comput Assist Tomogr.

[16] Fang ZJ, Zhang JY, He WM (2013). CT features of exophthalmos in Chinese subjects with thyroid-
associated ophthalmopathy. Int J Ophthalmol.

[17] Bartalena L, Tanda ML, Piantanida E (2004). Relationship between management of hyperthyroidism and course of
the ophthalmopathy. J Endocrinol Invest.

[18] Dawes L, Elfeky M, Bell D Extraocular muscle involvement in thyroid associated orbitopathy
(mnemonic). Reference article, Radiopaedia.org.

[19] Karhanová M, Kovář R, Fryšák Z (2014). Extraocular muscle involvement in patients with
thyroid-associated orbitopathy. Cesk Slov Oftalmol.

[20] Gonçalves ACP, Silva LN, Gebrim EMMS (2012). Predicting dysthyroid optic neuropathy using computed tomography
volumetric analyses of orbital structures. Clinics (Sao Paulo).

[21] Weis E, Heran MKS, Jhamb A (2012). Quantitative computed tomographic predictors of compressive optic
neuropathy in patients with thyroid orbitopathy: a volumetric
analysis. Ophthalmology.

[22] Szelog J, Swanson H, Sniegowski MC (2022). Thyroid eye disease. Mo Med.

[23] Soroudi AE, Goldberg RA, McCann JD (2004). Prevalence of asymmetric exophthalmos in Graves
orbitopathy. Ophthalmic Plast Reconstr Surg.

[24] Daumerie C, Duprez T, Boschi A (2008). Long-term multidisciplinary follow-up of unilateral
thyroid-associated orbitopathy. Eur J Internal Med.

[25] Wiersinga WM, Smit T, van der Gaag R (1989). Clinical presentation of Graves’ ophthalmopathy. Ophthalmic Res.

[26] Bartley GB (1994). The epidemiologic characteristics and clinical course of
ophthalmopathy associated with autoimmune thyroid disease in Olmsted County,
Minnesota. Trans Am Ophthalmol Soc.

[27] Prummel MF, Bakker A, Wiersinga WM (2003). Multi-center study on the characteristics and treatment
strategies of patients with Graves’ orbitopathy: the first European Group on
Graves’ Orbitopathy experience. Eur J Endocrinol.

[28] Ponto KA, Binder H, Diana T (2015). Prevalence, phenotype, and psychosocial well-being in
euthyroid/hypothyroid thyroid-associated orbitopathy. Thyroid.

[29] Wiersinga WM, Bleumink M, Saeed P (2008). Is sleeping position related to asymmetry in bilateral Graves’
ophthalmopathy?. Thyroid.

[30] Panagiotou G, Perros P (2020). Asymmetric Graves’ orbitopathy. Front Endocrinol (Lausanne).

[31] Strianese D, Piscopo R, Elefante A (2013). Unilateral proptosis in thyroid eye disease with subsequent
contralateral involvement: retrospective follow-up study. BMC Ophthalmol.

[32] Garrity JA, Bahn RS (2006). Pathogenesis of Graves ophthalmopathy: implications for
prediction, prevention, and treatment. Am J Ophthalmol.

[33] Regensburg NI, Wiersinga WM, Berendschot TTJM (2011). Do subtypes of Graves’ orbitopathy exist?. Ophthalmology.

[34] Abreu EB (2011). Espessamento do tendão muscular na oftalmopatia de
Graves. Rev Bras Oftalmol.

